# Supplementary material for: Comparison of structural MRI brain measures between 1.5 and 3 T: Data from the Lothian Birth Cohort 1936
Source: Hum Brain Mapp. 2021 May 19;42(12):3905–21. doi: 10.1002/hbm.25473 (PMC8288101; doi:10.1002/hbm.25473)
Supplement: Supplementary file 1 — Appendix S1: Supporting Information [file HBM-42-3905-s001.docx]

Supplementary material for “Comparison of structural MRI brain measures between 1.5T and 3T: data from the Lothian Birth Cohort 1936”

Colin R. Buchanan, Susana Muñoz Maniega, Maria C. Valdés Hernández, Lucia Ballerini, Gayle Barclay, Adele M. Taylor, Tom C. Russ, Elliot M. Tucker-Drob, Joanna M. Wardlaw, Ian J. Deary, Mark E. Bastin, Simon R. Cox

# Tables

|  | **1.5T mean volume (SD)** | | **3.0T mean volume (SD)** | | **BSD** | **BSD** | **ICC** | **slope** | **intercept** | **predicted R^2^** |
| --- | --- | --- | --- | --- | --- | --- | --- | --- | --- | --- |
|  | **cm^3^** | | **cm^3^** | | **cm^3^** | **%** |  |  |  |  |
| **BrainSegVol** | 1001.684 | (100.472) | 1075.238 | (110.912) | 73.554 | 7.3 | 0.957 | 0.871 | 64.788 | 0.914 |
| **BrainSegVolNotVent** | 943.274 | (86.948) | 1019.201 | (96.456) | 75.927 | 8.1 | 0.939 | 0.851 | 75.827 | 0.860 |
| **BrainSegVolNotVentSurf** | 942.889 | (86.988) | 1018.771 | (96.469) | 75.882 | 8.1 | 0.938 | 0.851 | 76.403 | 0.859 |
| **lhCortexVol** | 196.002 | (15.747) | 215.476 | (18.300) | 19.474 | 10.0 | 0.880 | 0.765 | 31.073 | 0.759 |
| **rhCortexVol** | 196.926 | (15.874) | 217.330 | (17.655) | 20.404 | 10.4 | 0.890 | 0.805 | 21.999 | 0.798 |
| **CortexVol** | 392.928 | (31.355) | 432.806 | (35.802) | 39.878 | 10.2 | 0.892 | 0.788 | 51.808 | 0.779 |
| **lhCerebralWhiteMatterVol** | 193.298 | (24.271) | 206.770 | (29.931) | 13.472 | 7.1 | 0.802 | 0.665 | 55.858 | 0.615 |
| **rhCerebralWhiteMatterVol** | 193.592 | (24.548) | 206.839 | (29.810) | 13.247 | 6.9 | 0.837 | 0.702 | 48.408 | 0.676 |
| **CerebralWhiteMatterVol** | 386.890 | (48.602) | 413.609 | (59.439) | 26.719 | 7.0 | 0.824 | 0.688 | 102.418 | 0.704 |
| **SubCortGrayVol** | 46.056 | (4.989) | 48.920 | (4.598) | 2.864 | 6.6 | 0.851 | 0.926 | 0.733 | 0.752 |
| **TotalGrayVol** | 535.118 | (41.832) | 582.489 | (46.597) | 47.370 | 8.9 | 0.915 | 0.827 | 53.681 | 0.869 |
| **SupraTentorialVol** | 880.575 | (90.948) | 948.232 | (101.587) | 67.657 | 7.7 | 0.953 | 0.859 | 66.305 | 0.910 |
| **SupraTentorialVolNotVent** | 827.823 | (78.434) | 897.860 | (88.184) | 70.037 | 8.5 | 0.933 | 0.836 | 77.513 | 0.858 |
| **SupraTentorialVolNotVentVox** | 826.144 | (78.102) | 895.620 | (87.757) | 69.476 | 8.4 | 0.934 | 0.837 | 76.475 | 0.858 |
| **MaskVol** | 1447.730 | (139.710) | 1602.763 | (169.854) | 155.033 | 10.8 | 0.837 | 0.702 | 322.742 | 0.786 |
| **EstimatedTotalIntraCranialVol** | 1411.835 | (249.336) | 1469.029 | (237.716) | 57.193 | 4.8 | 0.680 | 0.714 | 363.028 | 0.575 |
| **TotalVentricularCSFVol** | 57.283 | (25.133) | 54.932 | (23.992) | -2.351 | -4.1 | 0.993 | 1.041 | 0.087 | 0.991 |
| **TotalCSFVol** | 58.855 | (25.636) | 56.239 | (24.319) | -2.616 | -4.4 | 0.993 | 1.048 | -0.086 | 0.991 |

**Supplementary Table 1.** Between-scanner statistics for the global volumetric measures estimated by the FreeSurfer volumetric processing stream for 91 participants scanned at both 1.5T and 3.0T: mean values, between-scanner differences (BSD), intraclass correlation coefficients (ICC), slopes and intercepts estimated by a linear model.

|  | **1.5T mean volume (SD)** | | **3.0T mean volume (SD)** | | **BSD** | **BSD** | **ICC** | **slope** | **intercept** | **predicted R^2^** |
| --- | --- | --- | --- | --- | --- | --- | --- | --- | --- | --- |
|  | **cm^3^** | | **cm^3^** | | **cm^3^** | **%** |  |  |  |  |
| **Left.Lateral.Ventricle** | 26.830 | (12.885) | 25.111 | (12.166) | -1.719 | -6.5 | 0.987 | 1.047 | 0.542 | 0.986 |
| **Left.Inf.Lat.Vent** | 1.060 | (0.656) | 0.940 | (0.593) | -0.120 | -9.9 | 0.958 | 1.066 | 0.058 | 0.912 |
| **Left.Cerebellum.White.Matter** | 11.166 | (1.604) | 12.060 | (2.096) | 0.893 | 9.1 | 0.455 | 0.361 | 6.816 | 0.325 |
| **Left.Cerebellum.Cortex** | 47.786 | (5.861) | 49.178 | (5.274) | 1.392 | 3.3 | 0.856 | 0.957 | 0.719 | 0.736 |
| **Left.Thalamus.Proper** | 5.853 | (0.685) | 6.533 | (0.784) | 0.680 | 11.8 | 0.853 | 0.751 | 0.946 | 0.823 |
| **Left.Caudate** | 3.334 | (0.544) | 3.381 | (0.576) | 0.047 | 2.0 | 0.744 | 0.704 | 0.955 | 0.669 |
| **Left.Putamen** | 3.826 | (0.902) | 3.999 | (0.579) | 0.173 | 9.3 | 0.602 | 1.031 | -0.297 | 0.465 |
| **Left.Pallidum** | 1.463 | (0.281) | 1.595 | (0.257) | 0.132 | 13.1 | 0.303 | 0.334 | 0.930 | 0.130 |
| **X3rd.Ventricle** | 2.142 | (0.594) | 2.207 | (0.757) | 0.065 | 2.2 | 0.853 | 0.690 | 0.620 | 0.798 |
| **X4th.Ventricle** | 1.854 | (0.670) | 2.092 | (0.668) | 0.237 | 16.0 | 0.937 | 0.939 | -0.111 | 0.872 |
| **Brain.Stem** | 18.589 | (2.051) | 22.526 | (3.640) | 3.936 | 21.0 | 0.685 | 0.451 | 8.431 | 0.658 |
| **Left.Hippocampus** | 2.975 | (0.456) | 3.104 | (0.424) | 0.129 | 6.2 | 0.607 | 0.656 | 0.938 | 0.544 |
| **Left.Amygdala** | 1.001 | (0.260) | 1.092 | (0.186) | 0.091 | 18.0 | 0.551 | 0.809 | 0.117 | 0.422 |
| **CSF** | 1.573 | (0.686) | 1.308 | (0.469) | -0.265 | -12.8 | 0.788 | 1.238 | -0.047 | 0.692 |
| **Left.Accumbens.area** | 0.433 | (0.080) | 0.337 | (0.090) | -0.096 | -21.0 | 0.434 | 0.390 | 0.301 | 0.240 |
| **Left.VentralDC** | 2.969 | (0.334) | 3.783 | (0.602) | 0.814 | 27.6 | 0.525 | 0.343 | 1.671 | 0.385 |
| **Left.choroid.plexus** | 0.560 | (0.153) | 0.944 | (0.321) | 0.383 | 78.2 | 0.198 | 0.122 | 0.445 | 0.216 |
| **Right.Lateral.Ventricle** | 23.264 | (11.365) | 21.781 | (10.257) | -1.483 | -6.0 | 0.985 | 1.097 | -0.623 | 0.990 |
| **Right.Inf.Lat.Vent** | 0.972 | (0.613) | 0.890 | (0.549) | -0.081 | -5.1 | 0.942 | 1.059 | 0.029 | 0.858 |
| **Right.Cerebellum.White.Matter** | 11.074 | (1.588) | 11.552 | (1.922) | 0.478 | 6.0 | 0.348 | 0.293 | 7.690 | 0.330 |
| **Right.Cerebellum.Cortex** | 47.776 | (5.445) | 50.258 | (5.661) | 2.482 | 5.5 | 0.822 | 0.792 | 7.988 | 0.745 |
| **Right.Thalamus.Proper** | 5.728 | (0.587) | 6.160 | (0.670) | 0.432 | 7.7 | 0.799 | 0.706 | 1.378 | 0.651 |
| **Right.Caudate** | 3.524 | (0.609) | 3.472 | (0.560) | -0.052 | -0.7 | 0.760 | 0.829 | 0.646 | 0.648 |
| **Right.Putamen** | 3.857 | (0.888) | 4.089 | (0.493) | 0.232 | 11.0 | 0.526 | 1.115 | -0.703 | 0.456 |
| **Right.Pallidum** | 1.478 | (0.233) | 1.707 | (0.283) | 0.229 | 18.0 | 0.217 | 0.182 | 1.167 | 0.131 |
| **Right.Hippocampus** | 3.063 | (0.418) | 3.119 | (0.359) | 0.056 | 2.6 | 0.765 | 0.902 | 0.249 | 0.601 |
| **Right.Amygdala** | 1.252 | (0.261) | 1.272 | (0.185) | 0.021 | 5.3 | 0.622 | 0.926 | 0.073 | 0.494 |
| **Right.Accumbens.area** | 0.436 | (0.087) | 0.384 | (0.078) | -0.052 | -10.7 | 0.604 | 0.671 | 0.179 | 0.441 |
| **Right.VentralDC** | 2.965 | (0.337) | 3.472 | (0.576) | 0.507 | 17.2 | 0.555 | 0.373 | 1.671 | 0.462 |
| **Right.choroid.plexus** | 0.600 | (0.151) | 0.966 | (0.352) | 0.366 | 71.3 | 0.142 | 0.084 | 0.519 | 0.226 |
| **Optic.Chiasm** | 0.220 | (0.045) | 0.197 | (0.041) | -0.022 | -5.8 | 0.239 | 0.262 | 0.168 | 0.143 |
| **CC_Posterior** | 0.875 | (0.156) | 0.998 | (0.199) | 0.124 | 18.1 | 0.238 | 0.192 | 0.683 | 0.278 |
| **CC_Mid_Posterior** | 0.349 | (0.072) | 0.546 | (0.180) | 0.196 | 59.5 | 0.216 | 0.125 | 0.281 | 0.156 |
| **CC_Central** | 0.361 | (0.056) | 0.528 | (0.149) | 0.168 | 49.0 | 0.138 | 0.079 | 0.319 | 0.222 |
| **CC_Mid_Anterior** | 0.357 | (0.064) | 0.499 | (0.165) | 0.142 | 41.4 | 0.243 | 0.140 | 0.287 | 0.381 |
| **CC_Anterior** | 0.748 | (0.134) | 0.896 | (0.152) | 0.148 | 21.5 | 0.645 | 0.575 | 0.233 | 0.591 |

**Supplementary Table 2.** Between-scanner statistics for the subcortical, ventricular and white matter volumetric measures estimated by the FreeSurfer volumetric processing stream for 91 participants scanned at both 1.5T and 3.0T: mean values, between-scanner differences (BSD), intraclass correlation coefficients (ICC), slopes and intercepts estimated by a linear model.

|  | **1.5T mean volume (SD)** | | **3.0T mean volume (SD)** | | **BSD** | **BSD** | **ICC** | **slope** | **intercept** | **predicted R^2^** |
| --- | --- | --- | --- | --- | --- | --- | --- | --- | --- | --- |
|  | **cm^3^** | | **cm^3^** | | **cm^3^** | **%** |  |  |  |  |
| **L-Banks of superior temporal** | 1.904 | (0.336) | 2.266 | (0.390) | 0.363 | 19.9 | 0.762 | 0.664 | 0.399 | 0.611 |
| **L-Caudal anterior cingulate** | 1.285 | (0.343) | 1.627 | (0.443) | 0.342 | 28.6 | 0.789 | 0.631 | 0.259 | 0.753 |
| **L-Caudal middle frontal** | 5.176 | (0.726) | 5.844 | (0.834) | 0.668 | 13.4 | 0.781 | 0.687 | 1.164 | 0.643 |
| **L-Cuneus** | 2.632 | (0.424) | 2.977 | (0.464) | 0.345 | 13.5 | 0.864 | 0.792 | 0.273 | 0.760 |
| **L-Entorhinal** | 1.721 | (0.489) | 1.602 | (0.327) | -0.119 | 1.0 | 0.461 | 0.747 | 0.525 | 0.390 |
| **L-Fusiform** | 8.219 | (1.161) | 8.665 | (1.268) | 0.446 | 5.8 | 0.808 | 0.743 | 1.781 | 0.680 |
| **L-Inferior parietal** | 9.686 | (1.386) | 10.838 | (1.491) | 1.152 | 12.2 | 0.908 | 0.847 | 0.511 | 0.854 |
| **L-Inferior temporal** | 8.971 | (1.435) | 10.007 | (1.621) | 1.036 | 12.0 | 0.830 | 0.740 | 1.562 | 0.740 |
| **L-Isthmus cingulate** | 2.294 | (0.303) | 2.210 | (0.332) | -0.085 | -3.4 | 0.682 | 0.625 | 0.913 | 0.558 |
| **L-Lateral occipital** | 10.364 | (1.479) | 11.475 | (1.687) | 1.111 | 10.9 | 0.884 | 0.781 | 1.398 | 0.815 |
| **L-Lateral orbitofrontal** | 6.379 | (0.799) | 7.201 | (0.706) | 0.822 | 13.6 | 0.636 | 0.725 | 1.159 | 0.525 |
| **L-Lingual** | 5.635 | (0.747) | 6.019 | (0.766) | 0.384 | 7.1 | 0.892 | 0.871 | 0.395 | 0.824 |
| **L-Medial orbitofrontal** | 4.562 | (0.567) | 5.231 | (0.540) | 0.669 | 15.7 | 0.501 | 0.527 | 1.806 | 0.361 |
| **L-Middle temporal** | 8.432 | (1.238) | 9.452 | (1.403) | 1.020 | 12.5 | 0.851 | 0.756 | 1.283 | 0.698 |
| **L-Parahippocampal** | 1.747 | (0.324) | 1.797 | (0.297) | 0.050 | 4.3 | 0.649 | 0.710 | 0.471 | 0.520 |
| **L-Paracentral** | 3.251 | (0.371) | 3.335 | (0.464) | 0.084 | 2.7 | 0.722 | 0.591 | 1.279 | 0.547 |
| **L-Pars opercularis** | 3.668 | (0.507) | 4.235 | (0.592) | 0.567 | 15.9 | 0.810 | 0.702 | 0.695 | 0.735 |
| **L-Pars orbitalis** | 1.970 | (0.322) | 2.080 | (0.284) | 0.111 | 6.7 | 0.626 | 0.714 | 0.485 | 0.477 |
| **L-Pars triangularis** | 2.819 | (0.365) | 3.203 | (0.456) | 0.384 | 13.8 | 0.796 | 0.653 | 0.729 | 0.676 |
| **L-Pericalcarine** | 1.834 | (0.381) | 2.157 | (0.376) | 0.322 | 19.1 | 0.791 | 0.802 | 0.105 | 0.627 |
| **L-Postcentral** | 8.479 | (0.936) | 9.011 | (1.115) | 0.532 | 6.4 | 0.757 | 0.645 | 2.666 | 0.623 |
| **L-Posterior cingulate** | 2.640 | (0.397) | 2.783 | (0.497) | 0.144 | 5.7 | 0.743 | 0.609 | 0.946 | 0.594 |
| **L-Precentral** | 11.457 | (1.126) | 11.847 | (1.523) | 0.390 | 3.5 | 0.674 | 0.521 | 5.283 | 0.584 |
| **L-Precuneus** | 8.032 | (0.890) | 9.129 | (1.057) | 1.097 | 13.8 | 0.833 | 0.712 | 1.535 | 0.761 |
| **L-Rostral anterior cingulate** | 2.037 | (0.424) | 2.486 | (0.434) | 0.448 | 25.9 | 0.568 | 0.554 | 0.660 | 0.465 |
| **L-Rostral middle frontal** | 11.580 | (1.607) | 14.099 | (2.099) | 2.519 | 21.9 | 0.857 | 0.680 | 1.997 | 0.823 |
| **L-Superior frontal** | 18.686 | (1.846) | 20.264 | (2.089) | 1.578 | 8.6 | 0.836 | 0.745 | 3.599 | 0.657 |
| **L-Superior parietal** | 11.050 | (1.497) | 12.442 | (1.597) | 1.392 | 13.0 | 0.850 | 0.799 | 1.115 | 0.732 |
| **L-Superior temporal** | 9.727 | (1.298) | 11.022 | (1.381) | 1.295 | 13.8 | 0.854 | 0.804 | 0.865 | 0.777 |
| **L-Supramarginal** | 9.361 | (1.264) | 10.372 | (1.580) | 1.012 | 10.8 | 0.890 | 0.730 | 1.794 | 0.791 |
| **L-Frontal pole** | 0.887 | (0.164) | 0.915 | (0.133) | 0.028 | 5.5 | 0.340 | 0.429 | 0.495 | 0.283 |
| **L-Temporal pole** | 2.364 | (0.486) | 2.250 | (0.442) | -0.114 | -2.7 | 0.499 | 0.551 | 1.125 | 0.357 |
| **L-Transverse temporal** | 0.987 | (0.150) | 1.098 | (0.173) | 0.111 | 11.7 | 0.793 | 0.697 | 0.222 | 0.622 |
| **L-Insula** | 6.800 | (0.862) | 6.674 | (0.686) | -0.126 | -1.2 | 0.639 | 0.824 | 1.298 | 0.463 |
| **R-Banks of superior temporal** | 1.706 | (0.262) | 2.132 | (0.325) | 0.426 | 25.5 | 0.810 | 0.668 | 0.283 | 0.671 |
| **R-Caudal anterior cingulate** | 1.585 | (0.393) | 1.880 | (0.445) | 0.295 | 19.9 | 0.886 | 0.788 | 0.103 | 0.818 |
| **R-Caudal middle frontal** | 4.944 | (0.683) | 5.513 | (0.863) | 0.569 | 11.5 | 0.858 | 0.698 | 1.098 | 0.805 |
| **R-Cuneus** | 2.841 | (0.481) | 3.233 | (0.519) | 0.391 | 14.3 | 0.836 | 0.777 | 0.329 | 0.751 |
| **R-Entorhinal** | 1.800 | (0.442) | 1.617 | (0.394) | -0.183 | -5.4 | 0.454 | 0.513 | 0.971 | 0.279 |
| **R-Fusiform** | 7.886 | (0.966) | 8.305 | (0.939) | 0.419 | 5.9 | 0.686 | 0.706 | 2.021 | 0.520 |
| **R-Inferior parietal** | 11.635 | (1.683) | 13.030 | (1.851) | 1.395 | 12.3 | 0.882 | 0.806 | 1.136 | 0.818 |
| **R-Inferior temporal** | 8.685 | (1.297) | 9.708 | (1.391) | 1.023 | 12.3 | 0.819 | 0.766 | 1.251 | 0.694 |
| **R-Isthmus cingulate** | 2.110 | (0.301) | 2.077 | (0.342) | -0.033 | -1.3 | 0.733 | 0.650 | 0.761 | 0.605 |
| **R-Lateral occipital** | 10.574 | (1.583) | 11.591 | (1.690) | 1.017 | 9.9 | 0.895 | 0.840 | 0.841 | 0.823 |
| **R-Lateral orbitofrontal** | 6.172 | (0.748) | 7.081 | (0.718) | 0.909 | 15.3 | 0.718 | 0.749 | 0.866 | 0.547 |
| **R-Lingual** | 6.160 | (0.996) | 6.418 | (0.951) | 0.258 | 4.6 | 0.917 | 0.962 | -0.012 | 0.850 |
| **R-Medial orbitofrontal** | 4.772 | (0.516) | 5.659 | (0.525) | 0.887 | 19.1 | 0.708 | 0.696 | 0.836 | 0.546 |
| **R-Middle temporal** | 9.394 | (1.120) | 10.475 | (1.201) | 1.082 | 11.8 | 0.834 | 0.780 | 1.224 | 0.712 |
| **R-Parahippocampal** | 1.650 | (0.258) | 1.778 | (0.243) | 0.127 | 8.9 | 0.676 | 0.718 | 0.374 | 0.498 |
| **R-Paracentral** | 3.526 | (0.388) | 3.646 | (0.511) | 0.120 | 3.3 | 0.795 | 0.627 | 1.242 | 0.694 |
| **R-Pars opercularis** | 3.178 | (0.445) | 3.627 | (0.484) | 0.449 | 14.5 | 0.884 | 0.815 | 0.222 | 0.814 |
| **R-Pars orbitalis** | 2.284 | (0.328) | 2.479 | (0.331) | 0.195 | 9.2 | 0.662 | 0.657 | 0.656 | 0.521 |
| **R-Pars triangularis** | 3.288 | (0.508) | 3.764 | (0.610) | 0.476 | 14.6 | 0.895 | 0.757 | 0.437 | 0.846 |
| **R-Pericalcarine** | 2.023 | (0.361) | 2.384 | (0.420) | 0.362 | 18.8 | 0.753 | 0.655 | 0.461 | 0.620 |
| **R-Postcentral** | 8.107 | (0.974) | 8.607 | (1.101) | 0.499 | 6.4 | 0.782 | 0.697 | 2.108 | 0.642 |
| **R-Posterior cingulate** | 2.618 | (0.345) | 2.768 | (0.411) | 0.150 | 6.1 | 0.696 | 0.593 | 0.976 | 0.570 |
| **R-Precentral** | 11.262 | (1.194) | 11.819 | (1.464) | 0.557 | 5.1 | 0.708 | 0.589 | 4.296 | 0.548 |
| **R-Precuneus** | 8.380 | (0.956) | 9.574 | (1.103) | 1.194 | 14.3 | 0.921 | 0.806 | 0.659 | 0.860 |
| **R-Rostral anterior cingulate** | 1.548 | (0.366) | 1.820 | (0.408) | 0.272 | 19.5 | 0.796 | 0.719 | 0.239 | 0.678 |
| **R-Rostral middle frontal** | 12.081 | (1.834) | 14.838 | (2.249) | 2.758 | 23.0 | 0.895 | 0.746 | 1.019 | 0.811 |
| **R-Superior frontal** | 17.809 | (1.847) | 19.422 | (2.076) | 1.613 | 9.3 | 0.721 | 0.645 | 5.275 | 0.600 |
| **R-Superior parietal** | 10.759 | (1.461) | 12.210 | (1.524) | 1.451 | 14.1 | 0.750 | 0.719 | 1.979 | 0.615 |
| **R-Superior temporal** | 9.241 | (1.126) | 10.412 | (1.118) | 1.171 | 13.1 | 0.849 | 0.855 | 0.336 | 0.735 |
| **R-Supramarginal** | 8.476 | (1.104) | 9.387 | (1.207) | 0.912 | 11.1 | 0.832 | 0.764 | 1.305 | 0.738 |
| **R-Frontal pole** | 1.097 | (0.190) | 1.250 | (0.216) | 0.153 | 15.9 | 0.362 | 0.321 | 0.696 | 0.202 |
| **R-Temporal pole** | 2.538 | (0.544) | 2.300 | (0.429) | -0.237 | -7.0 | 0.436 | 0.568 | 1.231 | 0.278 |
| **R-Transverse temporal** | 0.793 | (0.134) | 0.873 | (0.140) | 0.079 | 11.1 | 0.695 | 0.668 | 0.211 | 0.551 |
| **R-Insula** | 6.380 | (0.678) | 6.575 | (0.632) | 0.195 | 3.5 | 0.687 | 0.739 | 1.523 | 0.531 |

**Supplementary Table 3.** Between-scanner statistics for the volumes of 68 cortical regions identified by FreeSurfer 6.0 for 91 participants scanned at both 1.5T and 3.0T: mean values, between-scanner differences (BSD), intraclass correlation coefficients (ICC), slopes and intercepts estimated by a linear model.

|  | **1.5T mean area (SD)** | | **3.0T mean area (SD)** | | **BSD** | **BSD** | **ICC** | **slope** | **intercept** | **predicted R^2^** |
| --- | --- | --- | --- | --- | --- | --- | --- | --- | --- | --- |
|  | **cm^2^** | | **cm^2^** | | **cm^2^** | **%** |  |  |  |  |
| **L-Banks of superior temporal** | 8.522 | (1.418) | 9.993 | (1.736) | 1.471 | 17.9 | 0.752 | 0.627 | 2.255 | 0.635 |
| **L-Caudal anterior cingulate** | 5.338 | (0.921) | 6.416 | (1.481) | 1.078 | 20.3 | 0.642 | 0.445 | 2.484 | 0.532 |
| **L-Caudal middle frontal** | 20.092 | (2.860) | 23.931 | (3.761) | 3.839 | 19.5 | 0.740 | 0.584 | 6.117 | 0.621 |
| **L-Cuneus** | 13.765 | (2.054) | 14.811 | (2.597) | 1.046 | 7.5 | 0.873 | 0.710 | 3.253 | 0.821 |
| **L-Entorhinal** | 3.843 | (0.827) | 3.766 | (0.817) | -0.077 | 0.6 | 0.386 | 0.391 | 2.371 | 0.297 |
| **L-Fusiform** | 26.649 | (3.956) | 29.080 | (4.496) | 2.431 | 9.6 | 0.763 | 0.676 | 6.979 | 0.611 |
| **L-Inferior parietal** | 38.084 | (5.347) | 43.691 | (6.940) | 5.607 | 14.8 | 0.846 | 0.674 | 8.640 | 0.780 |
| **L-Inferior temporal** | 27.389 | (4.385) | 30.370 | (5.142) | 2.981 | 11.9 | 0.621 | 0.536 | 11.114 | 0.491 |
| **L-Isthmus cingulate** | 9.330 | (1.275) | 9.854 | (1.458) | 0.525 | 5.7 | 0.891 | 0.786 | 1.580 | 0.845 |
| **L-Lateral occipital** | 44.807 | (6.103) | 49.607 | (7.472) | 4.800 | 10.8 | 0.863 | 0.719 | 9.132 | 0.787 |
| **L-Lateral orbitofrontal** | 21.593 | (3.062) | 24.676 | (3.462) | 3.082 | 15.6 | 0.401 | 0.357 | 12.783 | 0.315 |
| **L-Lingual** | 26.286 | (3.228) | 28.284 | (3.758) | 1.998 | 7.8 | 0.816 | 0.709 | 6.223 | 0.720 |
| **L-Medial orbitofrontal** | 15.932 | (2.317) | 16.936 | (2.481) | 1.004 | 7.4 | 0.539 | 0.504 | 7.390 | 0.410 |
| **L-Middle temporal** | 26.368 | (3.631) | 29.710 | (4.132) | 3.342 | 13.4 | 0.663 | 0.587 | 8.914 | 0.517 |
| **L-Parahippocampal** | 5.975 | (0.959) | 5.928 | (0.738) | -0.047 | 0.3 | 0.493 | 0.663 | 2.047 | 0.496 |
| **L-Paracentral** | 12.551 | (1.428) | 14.288 | (1.909) | 1.737 | 13.8 | 0.820 | 0.640 | 3.409 | 0.733 |
| **L-Pars opercularis** | 13.758 | (1.782) | 15.789 | (2.198) | 2.031 | 15.1 | 0.791 | 0.655 | 3.409 | 0.687 |
| **L-Pars orbitalis** | 5.958 | (1.012) | 6.531 | (1.011) | 0.573 | 10.4 | 0.702 | 0.702 | 1.372 | 0.666 |
| **L-Pars triangularis** | 11.207 | (1.454) | 12.676 | (1.756) | 1.469 | 13.4 | 0.735 | 0.619 | 3.358 | 0.663 |
| **L-Pericalcarine** | 12.715 | (2.219) | 13.798 | (2.429) | 1.083 | 8.8 | 0.908 | 0.833 | 1.220 | 0.848 |
| **L-Postcentral** | 39.035 | (4.205) | 43.292 | (4.924) | 4.258 | 10.9 | 0.889 | 0.769 | 5.760 | 0.846 |
| **L-Posterior cingulate** | 10.146 | (1.469) | 11.195 | (1.978) | 1.049 | 10.5 | 0.732 | 0.568 | 3.790 | 0.630 |
| **L-Precentral** | 45.864 | (4.532) | 52.340 | (5.794) | 6.476 | 14.1 | 0.855 | 0.689 | 9.810 | 0.777 |
| **L-Precuneus** | 33.211 | (3.828) | 38.239 | (4.882) | 5.028 | 15.2 | 0.831 | 0.671 | 7.546 | 0.749 |
| **L-Rostral anterior cingulate** | 6.492 | (1.452) | 8.220 | (1.818) | 1.727 | 29.5 | 0.548 | 0.449 | 2.805 | 0.432 |
| **L-Rostral middle frontal** | 45.500 | (7.227) | 58.458 | (10.770) | 12.958 | 29.1 | 0.650 | 0.471 | 17.940 | 0.578 |
| **L-Superior frontal** | 64.714 | (7.707) | 74.425 | (9.756) | 9.712 | 15.1 | 0.837 | 0.679 | 14.152 | 0.776 |
| **L-Superior parietal** | 48.508 | (5.601) | 54.872 | (6.856) | 6.364 | 13.2 | 0.866 | 0.722 | 8.868 | 0.781 |
| **L-Superior temporal** | 34.969 | (3.936) | 39.694 | (5.209) | 4.725 | 13.5 | 0.825 | 0.648 | 9.251 | 0.783 |
| **L-Supramarginal** | 36.384 | (5.750) | 41.303 | (6.776) | 4.919 | 13.6 | 0.930 | 0.800 | 3.357 | 0.839 |
| **L-Frontal pole** | 2.452 | (0.266) | 2.396 | (0.293) | -0.055 | -1.6 | 0.430 | 0.393 | 1.510 | 0.324 |
| **L-Temporal pole** | 4.653 | (0.600) | 4.085 | (0.671) | -0.567 | -11.6 | 0.494 | 0.445 | 2.836 | 0.326 |
| **L-Transverse temporal** | 3.959 | (0.626) | 4.539 | (0.739) | 0.580 | 15.1 | 0.816 | 0.701 | 0.778 | 0.651 |
| **L-Insula** | 23.406 | (2.923) | 25.848 | (3.605) | 2.442 | 11.1 | 0.504 | 0.417 | 12.616 | 0.339 |
| **R-Banks of superior temporal** | 7.512 | (1.112) | 9.256 | (1.365) | 1.744 | 23.8 | 0.761 | 0.633 | 1.656 | 0.666 |
| **R-Caudal anterior cingulate** | 6.089 | (1.048) | 7.143 | (1.446) | 1.054 | 17.3 | 0.803 | 0.612 | 1.715 | 0.747 |
| **R-Caudal middle frontal** | 19.255 | (2.608) | 22.004 | (3.355) | 2.749 | 14.4 | 0.797 | 0.639 | 5.188 | 0.692 |
| **R-Cuneus** | 14.527 | (2.111) | 16.081 | (2.639) | 1.553 | 10.7 | 0.858 | 0.703 | 3.216 | 0.805 |
| **R-Entorhinal** | 3.717 | (0.820) | 3.681 | (0.850) | -0.036 | 1.3 | 0.525 | 0.507 | 1.849 | 0.276 |
| **R-Fusiform** | 25.432 | (3.095) | 28.873 | (3.974) | 3.441 | 13.9 | 0.675 | 0.542 | 9.772 | 0.534 |
| **R-Inferior parietal** | 45.191 | (6.487) | 53.520 | (8.161) | 8.330 | 18.5 | 0.882 | 0.720 | 6.676 | 0.836 |
| **R-Inferior temporal** | 26.639 | (4.148) | 30.192 | (4.998) | 3.554 | 14.2 | 0.630 | 0.532 | 10.586 | 0.529 |
| **R-Isthmus cingulate** | 8.361 | (1.236) | 8.949 | (1.425) | 0.588 | 7.4 | 0.798 | 0.699 | 2.102 | 0.699 |
| **R-Lateral occipital** | 44.137 | (6.040) | 50.256 | (7.921) | 6.120 | 13.8 | 0.861 | 0.681 | 9.928 | 0.823 |
| **R-Lateral orbitofrontal** | 21.311 | (2.935) | 24.962 | (3.574) | 3.651 | 18.2 | 0.519 | 0.435 | 10.456 | 0.335 |
| **R-Lingual** | 28.114 | (3.834) | 30.681 | (4.819) | 2.567 | 9.1 | 0.878 | 0.717 | 6.108 | 0.837 |
| **R-Medial orbitofrontal** | 16.170 | (2.318) | 17.039 | (2.244) | 0.870 | 6.6 | 0.460 | 0.475 | 8.069 | 0.245 |
| **R-Middle temporal** | 29.346 | (3.748) | 33.445 | (4.179) | 4.099 | 14.5 | 0.710 | 0.641 | 7.917 | 0.557 |
| **R-Parahippocampal** | 5.676 | (0.590) | 5.988 | (0.775) | 0.312 | 5.6 | 0.714 | 0.564 | 2.299 | 0.583 |
| **R-Paracentral** | 13.760 | (1.794) | 15.800 | (2.177) | 2.040 | 15.0 | 0.833 | 0.700 | 2.706 | 0.725 |
| **R-Pars opercularis** | 12.008 | (1.658) | 13.420 | (1.875) | 1.412 | 11.9 | 0.878 | 0.783 | 1.506 | 0.816 |
| **R-Pars orbitalis** | 7.119 | (0.957) | 7.877 | (0.998) | 0.758 | 11.0 | 0.831 | 0.798 | 0.835 | 0.704 |
| **R-Pars triangularis** | 13.092 | (2.070) | 14.770 | (2.353) | 1.678 | 13.0 | 0.909 | 0.806 | 1.185 | 0.845 |
| **R-Pericalcarine** | 13.953 | (2.194) | 15.525 | (2.718) | 1.572 | 11.4 | 0.874 | 0.721 | 2.756 | 0.852 |
| **R-Postcentral** | 37.536 | (4.200) | 41.832 | (5.015) | 4.295 | 11.5 | 0.870 | 0.740 | 6.570 | 0.795 |
| **R-Posterior cingulate** | 10.051 | (1.332) | 11.078 | (1.764) | 1.027 | 10.4 | 0.756 | 0.594 | 3.471 | 0.630 |
| **R-Precentral** | 45.394 | (4.707) | 51.709 | (5.559) | 6.315 | 14.0 | 0.886 | 0.761 | 6.050 | 0.818 |
| **R-Precuneus** | 34.896 | (4.225) | 40.545 | (5.414) | 5.648 | 16.2 | 0.878 | 0.707 | 6.251 | 0.841 |
| **R-Rostral anterior cingulate** | 4.454 | (1.040) | 5.483 | (1.201) | 1.029 | 26.1 | 0.622 | 0.544 | 1.470 | 0.533 |
| **R-Rostral middle frontal** | 48.269 | (8.179) | 59.600 | (10.896) | 11.331 | 23.9 | 0.787 | 0.615 | 11.593 | 0.723 |
| **R-Superior frontal** | 61.617 | (7.173) | 70.265 | (9.814) | 8.647 | 14.0 | 0.803 | 0.616 | 18.332 | 0.738 |
| **R-Superior parietal** | 48.149 | (5.653) | 55.490 | (6.993) | 7.341 | 15.5 | 0.781 | 0.646 | 12.303 | 0.686 |
| **R-Superior temporal** | 32.536 | (3.358) | 37.454 | (4.431) | 4.917 | 15.2 | 0.749 | 0.590 | 10.441 | 0.620 |
| **R-Supramarginal** | 32.794 | (4.620) | 38.108 | (5.508) | 5.315 | 16.5 | 0.853 | 0.727 | 5.107 | 0.814 |
| **R-Frontal pole** | 3.093 | (0.379) | 3.103 | (0.390) | 0.011 | 1.1 | 0.476 | 0.463 | 1.656 | 0.331 |
| **R-Temporal pole** | 4.681 | (0.660) | 4.097 | (0.707) | -0.585 | -12.0 | 0.598 | 0.559 | 2.392 | 0.399 |
| **R-Transverse temporal** | 2.972 | (0.426) | 3.510 | (0.517) | 0.539 | 18.5 | 0.798 | 0.670 | 0.621 | 0.664 |
| **R-Insula** | 21.879 | (2.832) | 24.715 | (3.245) | 2.836 | 14.2 | 0.335 | 0.295 | 14.588 | 0.195 |

**Supplementary Table 4.** Between-scanner statistics for the surface area of 68 cortical regions identified by FreeSurfer 6.0 for 91 participants scanned at both 1.5T and 3.0T: mean values, between-scanner differences (BSD), intraclass correlation coefficients (ICC), slopes and intercepts estimated by a linear model.

|  | **1.5T mean thickness (SD)** | | **3.0T mean thickness (SD)** | | **BSD** | **BSD** | **ICC** | **slope** | **intercept** | **predicted R^2^** |
| --- | --- | --- | --- | --- | --- | --- | --- | --- | --- | --- |
|  | **mm** | | **mm** | | **mm** | **%** |  |  |  |  |
| **L-Banks of superior temporal** | 2.205 | (0.163) | 2.407 | (0.143) | 0.202 | 9.4 | 0.680 | 0.781 | 0.324 | 0.546 |
| **L-Caudal anterior cingulate** | 2.354 | (0.350) | 2.476 | (0.212) | 0.122 | 6.9 | 0.458 | 0.849 | 0.250 | 0.357 |
| **L-Caudal middle frontal** | 2.304 | (0.136) | 2.388 | (0.192) | 0.084 | 3.7 | 0.488 | 0.367 | 1.429 | 0.362 |
| **L-Cuneus** | 1.769 | (0.112) | 1.914 | (0.133) | 0.145 | 8.4 | 0.480 | 0.412 | 0.980 | 0.502 |
| **L-Entorhinal** | 2.905 | (0.477) | 3.076 | (0.335) | 0.170 | 8.3 | 0.354 | 0.534 | 1.263 | 0.270 |
| **L-Fusiform** | 2.504 | (0.159) | 2.577 | (0.139) | 0.073 | 3.1 | 0.574 | 0.664 | 0.791 | 0.399 |
| **L-Inferior parietal** | 2.221 | (0.141) | 2.349 | (0.116) | 0.128 | 6.0 | 0.652 | 0.802 | 0.336 | 0.528 |
| **L-Inferior temporal** | 2.578 | (0.176) | 2.714 | (0.141) | 0.136 | 5.5 | 0.607 | 0.778 | 0.468 | 0.433 |
| **L-Isthmus cingulate** | 2.220 | (0.190) | 2.121 | (0.172) | -0.099 | -4.1 | 0.537 | 0.598 | 0.951 | 0.349 |
| **L-Lateral occipital** | 2.035 | (0.120) | 2.130 | (0.128) | 0.095 | 4.8 | 0.694 | 0.654 | 0.642 | 0.542 |
| **L-Lateral orbitofrontal** | 2.498 | (0.167) | 2.613 | (0.158) | 0.115 | 5.0 | 0.285 | 0.301 | 1.711 | 0.238 |
| **L-Lingual** | 1.912 | (0.112) | 1.988 | (0.115) | 0.076 | 4.1 | 0.539 | 0.525 | 0.868 | 0.352 |
| **L-Medial orbitofrontal** | 2.457 | (0.212) | 2.572 | (0.199) | 0.116 | 5.2 | 0.293 | 0.312 | 1.654 | 0.155 |
| **L-Middle temporal** | 2.497 | (0.181) | 2.631 | (0.154) | 0.134 | 5.6 | 0.605 | 0.718 | 0.608 | 0.478 |
| **L-Parahippocampal** | 2.366 | (0.332) | 2.632 | (0.269) | 0.266 | 12.3 | 0.711 | 0.899 | 0.000 | 0.590 |
| **L-Paracentral** | 2.335 | (0.148) | 2.276 | (0.215) | -0.059 | -2.4 | 0.442 | 0.326 | 1.593 | 0.336 |
| **L-Pars opercularis** | 2.294 | (0.141) | 2.454 | (0.132) | 0.160 | 7.2 | 0.545 | 0.582 | 0.865 | 0.423 |
| **L-Pars orbitalis** | 2.445 | (0.183) | 2.555 | (0.190) | 0.109 | 4.8 | 0.410 | 0.393 | 1.441 | 0.268 |
| **L-Pars triangularis** | 2.167 | (0.141) | 2.340 | (0.163) | 0.173 | 8.2 | 0.617 | 0.539 | 0.905 | 0.434 |
| **L-Pericalcarine** | 1.558 | (0.132) | 1.726 | (0.117) | 0.167 | 11.4 | 0.142 | 0.162 | 1.280 | 0.182 |
| **L-Postcentral** | 1.921 | (0.101) | 1.983 | (0.151) | 0.062 | 3.3 | 0.547 | 0.396 | 1.135 | 0.430 |
| **L-Posterior cingulate** | 2.326 | (0.191) | 2.316 | (0.148) | -0.010 | -0.1 | 0.565 | 0.756 | 0.574 | 0.361 |
| **L-Precentral** | 2.309 | (0.154) | 2.274 | (0.237) | -0.035 | -1.5 | 0.532 | 0.379 | 1.447 | 0.360 |
| **L-Precuneus** | 2.174 | (0.134) | 2.283 | (0.128) | 0.109 | 5.2 | 0.624 | 0.652 | 0.686 | 0.563 |
| **L-Rostral anterior cingulate** | 2.673 | (0.293) | 2.692 | (0.247) | 0.019 | 1.4 | 0.607 | 0.731 | 0.706 | 0.437 |
| **L-Rostral middle frontal** | 2.142 | (0.136) | 2.253 | (0.136) | 0.111 | 5.5 | 0.343 | 0.344 | 1.367 | 0.328 |
| **L-Superior frontal** | 2.412 | (0.148) | 2.491 | (0.152) | 0.079 | 3.4 | 0.612 | 0.594 | 0.931 | 0.448 |
| **L-Superior parietal** | 1.993 | (0.142) | 2.136 | (0.144) | 0.143 | 7.4 | 0.665 | 0.654 | 0.595 | 0.504 |
| **L-Superior temporal** | 2.340 | (0.173) | 2.507 | (0.143) | 0.167 | 7.4 | 0.668 | 0.826 | 0.270 | 0.547 |
| **L-Supramarginal** | 2.255 | (0.141) | 2.376 | (0.133) | 0.121 | 5.5 | 0.648 | 0.687 | 0.623 | 0.507 |
| **L-Frontal pole** | 2.492 | (0.281) | 2.634 | (0.271) | 0.141 | 6.4 | 0.494 | 0.512 | 1.143 | 0.338 |
| **L-Temporal pole** | 3.226 | (0.323) | 3.385 | (0.301) | 0.160 | 5.6 | 0.458 | 0.493 | 1.557 | 0.309 |
| **L-Transverse temporal** | 2.222 | (0.204) | 2.221 | (0.200) | -0.001 | 0.3 | 0.587 | 0.600 | 0.889 | 0.428 |
| **L-Insula** | 2.751 | (0.192) | 2.649 | (0.180) | -0.102 | -3.4 | 0.246 | 0.264 | 2.053 | 0.162 |
| **R-Banks of superior temporal** | 2.293 | (0.180) | 2.468 | (0.157) | 0.176 | 7.9 | 0.700 | 0.814 | 0.285 | 0.561 |
| **R-Caudal anterior cingulate** | 2.317 | (0.246) | 2.358 | (0.208) | 0.041 | 2.3 | 0.582 | 0.698 | 0.672 | 0.382 |
| **R-Caudal middle frontal** | 2.279 | (0.140) | 2.395 | (0.142) | 0.116 | 5.3 | 0.525 | 0.516 | 1.044 | 0.383 |
| **R-Cuneus** | 1.788 | (0.117) | 1.900 | (0.139) | 0.112 | 6.4 | 0.537 | 0.460 | 0.914 | 0.381 |
| **R-Entorhinal** | 3.041 | (0.440) | 3.056 | (0.348) | 0.015 | 2.2 | 0.286 | 0.371 | 1.906 | 0.155 |
| **R-Fusiform** | 2.518 | (0.161) | 2.528 | (0.144) | 0.010 | 0.6 | 0.498 | 0.561 | 1.099 | 0.308 |
| **R-Inferior parietal** | 2.239 | (0.149) | 2.315 | (0.129) | 0.076 | 3.6 | 0.561 | 0.658 | 0.717 | 0.438 |
| **R-Inferior temporal** | 2.575 | (0.176) | 2.684 | (0.132) | 0.109 | 4.5 | 0.468 | 0.648 | 0.835 | 0.351 |
| **R-Isthmus cingulate** | 2.251 | (0.231) | 2.135 | (0.173) | -0.117 | -4.6 | 0.529 | 0.735 | 0.683 | 0.431 |
| **R-Lateral occipital** | 2.096 | (0.127) | 2.150 | (0.119) | 0.054 | 2.8 | 0.513 | 0.548 | 0.918 | 0.347 |
| **R-Lateral orbitofrontal** | 2.498 | (0.182) | 2.567 | (0.165) | 0.068 | 3.1 | 0.418 | 0.464 | 1.307 | 0.271 |
| **R-Lingual** | 1.949 | (0.114) | 1.984 | (0.136) | 0.035 | 1.9 | 0.469 | 0.399 | 1.157 | 0.327 |
| **R-Medial orbitofrontal** | 2.457 | (0.194) | 2.663 | (0.199) | 0.206 | 8.9 | 0.198 | 0.193 | 1.944 | 0.208 |
| **R-Middle temporal** | 2.553 | (0.165) | 2.655 | (0.149) | 0.102 | 4.2 | 0.575 | 0.638 | 0.859 | 0.395 |
| **R-Parahippocampal** | 2.349 | (0.283) | 2.590 | (0.223) | 0.241 | 11.2 | 0.554 | 0.725 | 0.471 | 0.434 |
| **R-Paracentral** | 2.308 | (0.141) | 2.269 | (0.199) | -0.039 | -1.5 | 0.406 | 0.305 | 1.615 | 0.300 |
| **R-Pars opercularis** | 2.328 | (0.139) | 2.483 | (0.115) | 0.155 | 6.9 | 0.616 | 0.760 | 0.441 | 0.411 |
| **R-Pars orbitalis** | 2.422 | (0.213) | 2.580 | (0.186) | 0.157 | 7.0 | 0.489 | 0.566 | 0.962 | 0.360 |
| **R-Pars triangularis** | 2.185 | (0.159) | 2.353 | (0.139) | 0.168 | 7.9 | 0.577 | 0.666 | 0.619 | 0.434 |
| **R-Pericalcarine** | 1.546 | (0.122) | 1.678 | (0.135) | 0.132 | 9.0 | 0.279 | 0.253 | 1.121 | 0.215 |
| **R-Postcentral** | 1.918 | (0.118) | 1.972 | (0.157) | 0.054 | 2.9 | 0.504 | 0.394 | 1.141 | 0.322 |
| **R-Posterior cingulate** | 2.247 | (0.154) | 2.268 | (0.157) | 0.022 | 1.2 | 0.413 | 0.405 | 1.329 | 0.238 |
| **R-Precentral** | 2.296 | (0.150) | 2.291 | (0.213) | -0.005 | -0.1 | 0.504 | 0.378 | 1.430 | 0.387 |
| **R-Precuneus** | 2.165 | (0.133) | 2.273 | (0.132) | 0.108 | 5.1 | 0.783 | 0.785 | 0.380 | 0.684 |
| **R-Rostral anterior cingulate** | 2.932 | (0.278) | 2.864 | (0.263) | -0.068 | -1.8 | 0.443 | 0.468 | 1.592 | 0.303 |
| **R-Rostral middle frontal** | 2.122 | (0.149) | 2.281 | (0.128) | 0.159 | 7.8 | 0.450 | 0.527 | 0.920 | 0.328 |
| **R-Superior frontal** | 2.398 | (0.132) | 2.500 | (0.142) | 0.102 | 4.4 | 0.504 | 0.471 | 1.222 | 0.447 |
| **R-Superior parietal** | 1.972 | (0.147) | 2.104 | (0.141) | 0.132 | 6.9 | 0.599 | 0.625 | 0.657 | 0.435 |
| **R-Superior temporal** | 2.393 | (0.154) | 2.523 | (0.130) | 0.129 | 5.6 | 0.628 | 0.754 | 0.491 | 0.480 |
| **R-Supramarginal** | 2.287 | (0.149) | 2.365 | (0.127) | 0.079 | 3.7 | 0.534 | 0.636 | 0.781 | 0.381 |
| **R-Frontal pole** | 2.485 | (0.236) | 2.747 | (0.307) | 0.262 | 11.2 | 0.290 | 0.231 | 1.850 | 0.227 |
| **R-Temporal pole** | 3.357 | (0.334) | 3.439 | (0.321) | 0.082 | 3.0 | 0.455 | 0.474 | 1.728 | 0.289 |
| **R-Transverse temporal** | 2.282 | (0.228) | 2.229 | (0.210) | -0.053 | -1.8 | 0.454 | 0.496 | 1.178 | 0.419 |
| **R-Insula** | 2.724 | (0.192) | 2.663 | (0.157) | -0.061 | -1.8 | 0.213 | 0.265 | 2.017 | 0.197 |

**Supplementary Table 5.** Between-scanner statistics for the thickness of 68 cortical regions identified by FreeSurfer 6.0 for 91 participants scanned at both 1.5T and 3.0T: mean values, between-scanner differences (BSD), intraclass correlation coefficients (ICC), slopes and intercepts estimated by a linear model.

|  |  | **1.5T mean (SD)** | | **3.0T mean (SD)** | | **BSD** | **BSD (%)** | **ICC** | **slope** | **intercept** | **predicted R^2^** |
| --- | --- | --- | --- | --- | --- | --- | --- | --- | --- | --- | --- |
| **White matter** | **AD** | 1.041 | (0.045) | 1.103 | (0.041) | 0.062 | 6.0 | 0.776 | 0.870 | 0.081 | 0.651 |
|  | **RD** | 0.653 | (0.043) | 0.566 | (0.042) | -0.086 | -13.2 | 0.882 | 0.918 | 0.133 | 0.818 |
|  | **MD** | 0.782 | (0.043) | 0.745 | (0.040) | -0.037 | -4.7 | 0.867 | 0.937 | 0.084 | 0.762 |
|  | **FA** | 0.312 | (0.019) | 0.415 | (0.023) | 0.103 | 33.0 | 0.740 | 0.624 | 0.053 | 0.648 |
| **Whole-brain** | **AD** | 1.321 | (0.065) | 1.356 | (0.063) | 0.036 | 2.7 | 0.911 | 0.933 | 0.055 | 0.859 |
|  | **RD** | 1.069 | (0.066) | 1.036 | (0.065) | -0.033 | -3.1 | 0.932 | 0.947 | 0.088 | 0.859 |
|  | **MD** | 1.153 | (0.065) | 1.143 | (0.064) | -0.010 | -0.9 | 0.927 | 0.943 | 0.075 | 0.863 |
|  | **FA** | 0.197 | (0.012) | 0.225 | (0.010) | 0.028 | 14.3 | 0.860 | 1.033 | -0.035 | 0.801 |

**Supplementary Table 6.** Between-scanner statistics of four dMRI measures, namely, axial diffusivity (AD), radial diffusivity (RD), mean diffusivity (MD) and fractional anisotropy (FA), measured in cerebral white matter and in the whole brain for 79 participants scanned at both 1.5T and 3.0T: mean values, between-scanner differences (BSD), intraclass correlation coefficients (ICC), slopes and intercepts estimated by a linear model. AD, RD and MD are measured in x10^-3^ mm^2^/s.

|  |  | **1.5T mean (SD)** | | **3.0T mean (SD)** | | **BSD** | **BSD (%)** | **ICC** | **slope** | **intercept** | **predicted R^2^** |
| --- | --- | --- | --- | --- | --- | --- | --- | --- | --- | --- | --- |
| **Genu** | **MD** | 0.854 | 0.078 | 0.759 | 0.054 | -0.096 | -10.8 | 0.463 | 0.708 | 0.317 | 0.332 |
| **Splenium** | **MD** | 0.957 | 0.175 | 0.800 | 0.095 | -0.162 | -15.1 | 0.282 | 0.674 | 0.421 | 0.347 |
| **Left arcuate** | **MD** | 0.716 | 0.069 | 0.674 | 0.056 | -0.043 | -5.8 | 0.873 | 1.077 | -0.009 | 0.845 |
| **Right arcuate** | **MD** | 0.704 | 0.070 | 0.680 | 0.060 | -0.024 | -3.2 | 0.881 | 1.049 | -0.009 | 0.789 |
| **Left ATR** | **MD** | 0.784 | 0.071 | 0.733 | 0.053 | -0.051 | -6.1 | 0.562 | 0.776 | 0.215 | 0.392 |
| **Right ATR** | **MD** | 0.814 | 0.094 | 0.792 | 0.080 | -0.021 | -1.9 | 0.485 | 0.579 | 0.355 | 0.322 |
| **Left cingulum** | **MD** | 0.685 | 0.051 | 0.677 | 0.031 | -0.008 | -0.9 | 0.541 | 1.000 | 0.008 | 0.406 |
| **Right cingulum** | **MD** | 0.675 | 0.043 | 0.681 | 0.028 | 0.005 | 1.0 | 0.552 | 0.914 | 0.053 | 0.405 |
| **Left uncinate** | **MD** | 0.818 | 0.056 | 0.753 | 0.039 | -0.066 | -7.9 | 0.595 | 0.867 | 0.166 | 0.447 |
| **Right uncinate** | **MD** | 0.817 | 0.051 | 0.757 | 0.037 | -0.059 | -7.0 | 0.654 | 0.981 | 0.073 | 0.533 |
| **Left ILF** | **MD** | 0.863 | 0.149 | 0.781 | 0.093 | -0.076 | -7.5 | 0.243 | 0.361 | 0.574 | 0.295 |
| **Right ILF** | **MD** | 0.832 | 0.091 | 0.788 | 0.098 | -0.043 | -4.8 | 0.635 | 0.593 | 0.363 | 0.429 |
|  | **gMD** | -0.102 | 0.955 | -0.064 | 0.979 | 0.044 | - | 0.850 | 0.831 | -0.055 | 0.754 |
| **Genu** | **FA** | 0.369 | 0.042 | 0.514 | 0.039 | 0.146 | 40.9 | 0.433 | 0.469 | 0.128 | 0.235 |
| **Splenium** | **FA** | 0.479 | 0.070 | 0.674 | 0.056 | 0.197 | 43.7 | 0.430 | 0.568 | 0.095 | 0.288 |
| **Left arcuate** | **FA** | 0.433 | 0.042 | 0.548 | 0.042 | 0.116 | 27.2 | 0.723 | 0.723 | 0.037 | 0.596 |
| **Right arcuate** | **FA** | 0.409 | 0.039 | 0.503 | 0.041 | 0.094 | 23.6 | 0.597 | 0.565 | 0.125 | 0.409 |
| **Left ATR** | **FA** | 0.342 | 0.036 | 0.448 | 0.033 | 0.106 | 31.8 | 0.494 | 0.551 | 0.095 | 0.328 |
| **Right ATR** | **FA** | 0.345 | 0.043 | 0.431 | 0.036 | 0.086 | 26.4 | 0.459 | 0.536 | 0.114 | 0.312 |
| **Left cingulum** | **FA** | 0.431 | 0.052 | 0.608 | 0.052 | 0.178 | 42.9 | 0.430 | 0.429 | 0.170 | 0.286 |
| **Right cingulum** | **FA** | 0.402 | 0.053 | 0.551 | 0.057 | 0.149 | 38.7 | 0.442 | 0.411 | 0.175 | 0.303 |
| **Left uncinate** | **FA** | 0.322 | 0.038 | 0.474 | 0.032 | 0.152 | 48.6 | 0.551 | 0.646 | 0.016 | 0.342 |
| **Right uncinate** | **FA** | 0.321 | 0.032 | 0.461 | 0.026 | 0.140 | 44.3 | 0.516 | 0.675 | 0.011 | 0.336 |
| **Left ILF** | **FA** | 0.367 | 0.061 | 0.514 | 0.056 | 0.146 | 43.2 | 0.182 | 0.191 | 0.270 | 0.196 |
| **Right ILF** | **FA** | 0.375 | 0.051 | 0.508 | 0.053 | 0.133 | 37.3 | 0.447 | 0.424 | 0.159 | 0.303 |
|  | **gFA** | 0.104 | 0.912 | 0.021 | 0.965 | -0.014 | - | 0.769 | 0.712 | 0.042 | 0.651 |

**Supplementary Table 7.** Between-scanner statistics of MD and FA measured in 12 major white matter tracts plus general factors (gMD and gFA) using 90 participants scanned at both 1.5T and 3.0T: mean values, between-scanner differences (BSD), intraclass correlation coefficients (ICC), slopes and intercepts estimated by a linear model. MD is measured in x10^-3^ mm^2^/s.

|  | **MD** | | **FA** | |
| --- | --- | --- | --- | --- |
|  | **1.5T** | **3.0T** | **1.5T** | **3.0T** |
|  | **PC1** | **PC1** | **PC1** | **PC1** |
| **Genu** | 0.638 | 0.779 | 0.655 | 0.613 |
| **Splenium** | 0.501 | 0.450 | 0.519 | 0.351 |
| **Left arcuate** | 0.825 | 0.849 | 0.663 | 0.659 |
| **Right arcuate** | 0.847 | 0.823 | 0.660 | 0.536 |
| **Left ATR** | 0.669 | 0.774 | 0.603 | 0.669 |
| **Right ATR** | 0.591 | 0.554 | 0.584 | 0.394 |
| **Left cingulum** | 0.690 | 0.838 | 0.677 | 0.592 |
| **Right cingulum** | 0.798 | 0.823 | 0.649 | 0.487 |
| **Left uncinate** | 0.680 | 0.885 | 0.550 | 0.548 |
| **Right uncinate** | 0.716 | 0.837 | 0.621 | 0.672 |
| **Left ILF** | 0.316 | 0.658 | 0.358 | 0.536 |
| **Right ILF** | 0.432 | 0.537 | 0.412 | 0.560 |

**Supplementary Table 8.** Loadings of the 12 white matter tracts on the first principal component (PC1) computed independently for MD and FA and field strength.

|  |  | **1.5T mean (SD)** | | **3.0T mean (SD)** | | **BSD** | **BSD (%)** | **ICC** | **slope** | **intercept** | **predicted R^2^** |
| --- | --- | --- | --- | --- | --- | --- | --- | --- | --- | --- | --- |
| **Mean edge weight** | **MD** | 7.01E-04 | 4.00E-05 | 7.33E-04 | 3.92E-05 | 3.21E-05 | 4.7 | 0.612 | 0.625 | 2.43E-04 | 0.483 |
| **Network efficiency** | **MD** | 4.92E-04 | 2.77E-05 | 4.93E-04 | 2.84E-05 | 1.24E-06 | 0.3 | 0.888 | 0.867 | 6.42E-05 | 0.798 |
| **Network clustering coefficient** | **MD** | 5.26E-04 | 2.90E-05 | 4.98E-04 | 2.65E-05 | -2.79E-05 | -5.3 | 0.883 | 0.971 | 4.24E-05 | 0.827 |
| **Mean edge weight** | **FA** | 0.350 | 0.029 | 0.484 | 0.023 | 0.134 | 39.1 | 0.680 | 0.875 | -0.074 | 0.545 |
| **Network efficiency** | **FA** | 0.248 | 0.015 | 0.328 | 0.013 | 0.080 | 32.6 | 0.794 | 0.931 | -0.058 | 0.725 |
| **Network clustering coefficient** | **FA** | 0.248 | 0.015 | 0.318 | 0.013 | 0.070 | 28.6 | 0.799 | 0.898 | -0.038 | 0.679 |

**Supplementary Table 9.** Between-scanner statistics for global properties of whole-brain structural networks using 85 nodes with connection strength weighted by both MD and FA and computed using 79 participants scanned at both 1.5 and 3.0T (networks were thresholded to retain the top 30% consistent connections across participants): mean values, between-scanner differences (BSD), intraclass correlation coefficients (ICC), slopes and intercepts estimated by a linear model. MD is measured in mm^2^/s.

| **Region** | **Abbreviation** | **Lobe/Grouping** |
| --- | --- | --- |
| Caudal middle frontal | CaMF | Frontal |
| Frontal pole | FPo | Frontal |
| Lateral orbitofrontal | LOF | Frontal |
| Medial orbitofrontal | MedOr | Frontal |
| Paracentral | PaC | Frontal |
| Pars opercularis | ParOp | Frontal |
| Pars orbitalis | ParOr | Frontal |
| Pars triangularis | ParTr | Frontal |
| Precentral | PrC | Frontal |
| Rostral middle frontal | RosMF | Frontal |
| Superior frontal | SupF | Frontal |
| Caudal anterior cingulate | CaACg | Cingulate |
| Isthmus cingulate | IsCg | Cingulate |
| Posterior cingulate | PosCg | Cingulate |
| Rostral anterior cingulate | RosACg | Cingulate |
| Insula | Ins | - |
| Banks of superior temporal | bSTS | Temporal |
| Entorhinal | Ent | Temporal |
| Fusiform | Fus | Temporal |
| Inferior temporal | InfT | Temporal |
| Middle temporal | MT | Temporal |
| Parahippocampal | PaHip | Temporal |
| Superior temporal | SupT | Temporal |
| Temporal pole | TPo | Temporal |
| Transverse temporal | TrT | Temporal |
| Inferior parietal | InfP | Parietal |
| Postcentral | PosC | Parietal |
| Precuneus | PrCun | Parietal |
| Superior parietal | SupP | Parietal |
| Supramarginal | SuMar | Parietal |
| Cuneus | Cun | Occipital |
| Lateral occipital | LOc | Occipital |
| Lingual | Lin | Occipital |
| Pericalcarine | PerCa | Occipital |
| Nucleus accumbens | NAcc | Subcortical |
| Amygdala | Amg | Subcortical |
| Caudate nucleus | CaN | Subcortical |
| Hippocampus | Hip | Subcortical |
| Pallidum | Pal | Subcortical |
| Putamen | Put | Subcortical |
| Thalamus | Tha | Subcortical |
| Ventral diencephalon | VDC | Subcortical |
| Brainstem | BSt | - |

**Supplementary Table 10.** List of neuroanatomical regions (Desikan-Killiany atlas) and abbreviations.

# Figures


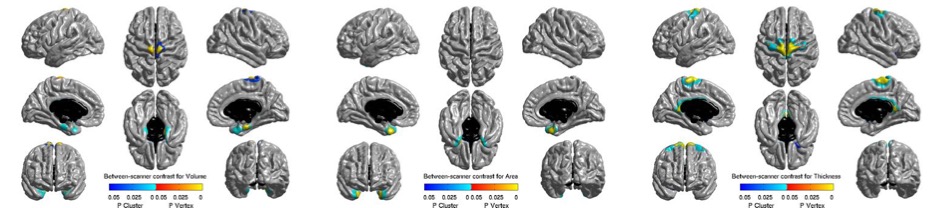


**Supplementary Figure 1.** Between-scanner contrasts for cortical volume, surface area and thickness for 91 participants imaged at both 1.5T and 3.0T. Heatmaps show the areas of significant difference between scanners (p < 0.05, uncorrected).


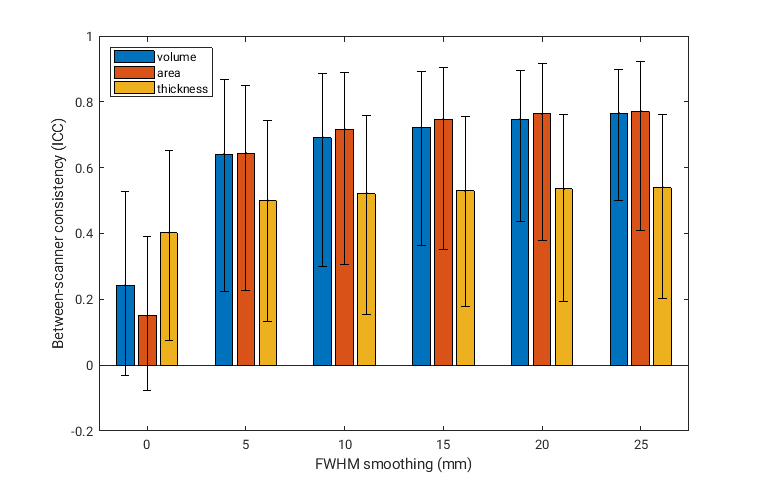


**Supplementary Figure 2.** Mean ICCs and 95% interpercentile range for cortical volume, surface area and thickness measured over six spatial FWHM smoothing levels (0 to 25 mm).


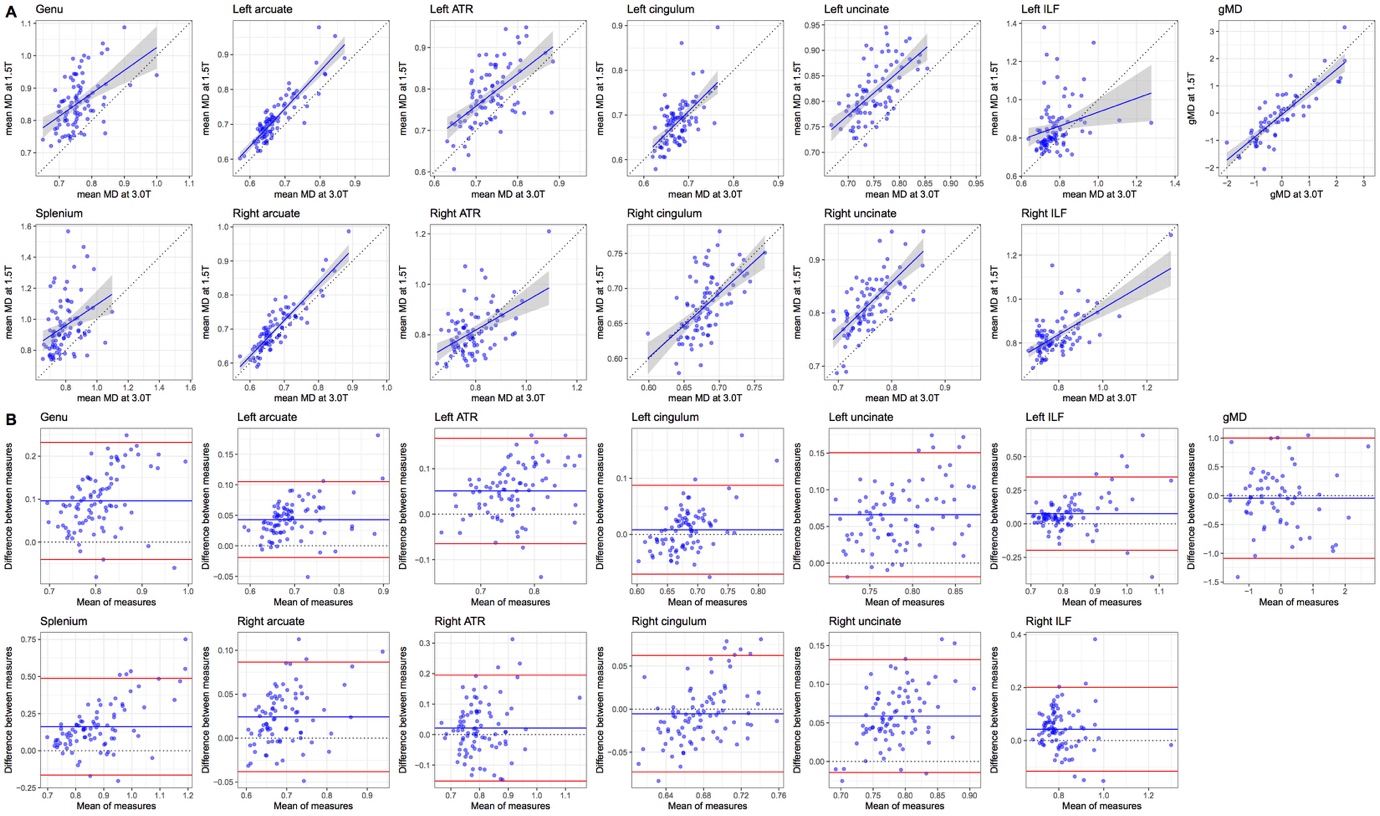


**Supplementary Figure 3.** Between-scanner differences of mean diffusivity (MD) measured in 12 white matter (plus an extracted general factor) for 79 participants scanned at both 1.5T and 3.0T: A) scatter plots where the continuous blue line shows linear fit with 95% CI. B) Bland-Altman plots of the same measures showing the mean of between-scanner measures and the difference between these measures where the blue line indicates the mean and the red lines represent ± 2 SDs.


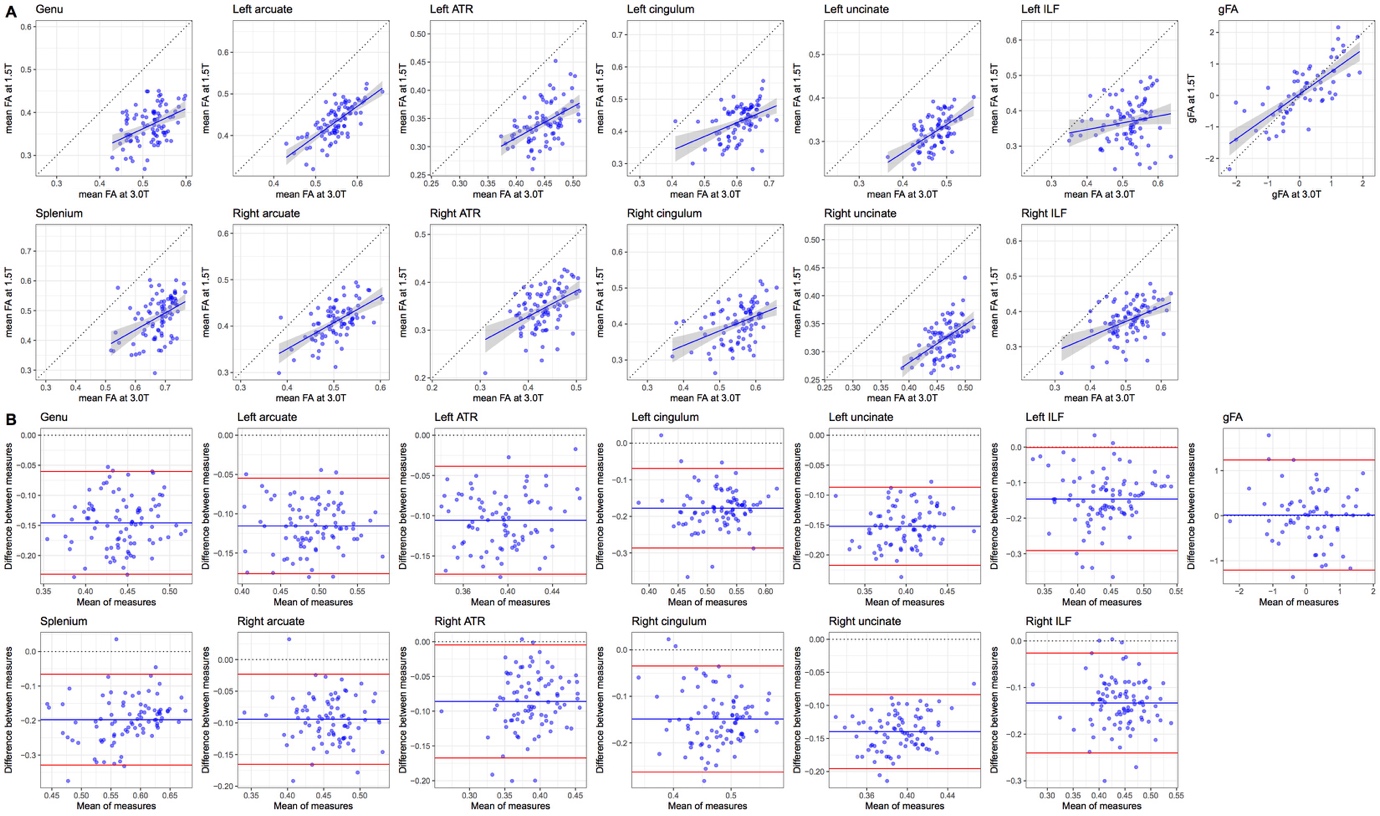


**Supplementary Figure 4.** Between-scanner differences of fractional anisotropy (FA), measured in 12 white matter (plus an extracted general factor) for 79 participants scanned at both 1.5T and 3.0T: A) scatter plots where the continuous blue line shows linear fit with 95% CI. B) Bland-Altman plots of the same measures showing the mean of between-scanner measures and the difference between these measures where the blue line indicates the mean and the red lines represent ± 2 SDs.

**Supplementary Figure 5.** Scatter plots showing all individual network weights (edges) pooled across all participants and measured at both 1.5T and 3.0T for MD-weighted (left) and FA-weighted networks (right). As networks are sparse, particularly at 1.5T, there are many zero-valued connections.
